# The Influence of Workplace Factors on the Nursing Work Environment: A Study Before and After COVID-19

**DOI:** 10.7759/cureus.26541

**Published:** 2022-07-03

**Authors:** Ahmad S Al Baker

**Affiliations:** 1 Department of Nursing, Sultan bin Abdulaziz Humanitarian City, Riyadh, SAU

**Keywords:** factors, workplace, covid-19, work environment, nursing

## Abstract

Introduction: The coronavirus disease 2019 (COVID-19) pandemic has impacted the world and healthcare settings, particularly regarding onerous job responsibilities and changes in work culture for nurses. Nurses, known to provide skilled, compassionate, and humanitarian care to patients and families, require continual assistance and organization. Nurses must work in a good environment that encourages them to achieve their highest levels of performance and productivity to offer high-quality and safe care. Commitment to a nursing career necessitates professional dedication especially during times of crisis, as is in the case of the COVID-19 pandemic.

Objectives: The study looked at the elements that influenced nursing work culture at Sultan bin Abdul Aziz Humanitarian City (SBAHC) Riyadh, Saudi Arabia during the COVID-19 epidemic. Leadership, satisfaction, teamwork, nurse behaviour in practice, and professional commitment were among these elements. The factors are interrelated together and help in shaping the nursing work culture considering the COVID-19 scenario.

Methods: A hospital-based cross-sectional study was conducted from January 2020 to December 2020, covering a period before the outbreak of COVID-19 and a period after. The study, conducted at SBAHC, Riyadh, Saudi Arabia, followed a quantitative, positivist approach undertaken in two phases utilizing an analytical survey design before and after the COVID-19 pandemic. An initial electronic survey was distributed to 439 nurses at SBAHC nursing units. Three hundred twenty-two nurses participated in the first survey and the second survey responses after COVID-19 were 205 nurses.

Results: There was a significant difference between the two groups in their responses (p<0.05). In all five scales used, the score of mean declined in the ‘after' group, clearly showing the effect of all the five elements that influence nursing work culture due to the COVID-19 pandemic.

Conclusion*:* The study concluded that the workplace factors influencing the nursing work environment were greatly affected due to the COVID-19 outbreak in Saudi Arabia. The findings of this study should be considered by nurse managers and leaders when drafting the policies and programs to reduce the negative impact of COVID-19 on nurse retention. It should also provide baseline information that will allow health authorities to prioritize training programs that will support nurses during difficult times.

## Introduction

The COVID-19 epidemic has had a significant impact on all healthcare professionals, particularly nurses. Health-care workers have faced increased workloads, longer working hours, a more stressful working environment, and sometimes a lack of medical and safety equipment during this time [1]. During the epidemic, healthcare workers have continued to try to balance their job and family obligations. Various physiological and psychological negative situations encountered by health care professionals as a result of the COVID-19 pandemic have been shown to increase their perception of organizational barriers and decrease their professional commitment [2].

There are many complicated work environments in the healthcare sector that are governed by strategies, systems, policies, and work norms, which is leading to the development of multi-focus areas of intentions and implementation solutions to support the crucial domain of healthy workplace cultures [3]. Nursing is a profession that necessitates a high level of professional dedication as well as a commitment to lifelong learning. Nurses who are dedicated to their profession will shape the future of nursing. Nurses must demonstrate a strong professional commitment to protecting patients' rights and do their jobs well thus it becomes essential to examine the factors affecting nurses' professional commitment during the pandemic [4].

In Saudi Arabia, the hospital operation system is divided based on ownership, each owner has their system of work policies and work practices which has a direct and indirect impact on nursing units [5]. Sultan bin Abdul Aziz Humanitarian City (SBAHC) is a private center that provides services in rehabilitation, in addition to surgical and medical needs. Frontline staff nurses and nurse assistants are the direct providers in collaboration with the interdisciplinary team. At SBAHC, the nursing profession faced challenges in work practice during the pandemic. Nurses, who are generally subjected to perform duties in a multidisciplinary and multinationalism framework found it stressful to continue with the same pace as before, during the pandemic. As a result, there is frustration and dissatisfaction in the workplace culture. This has a direct impact on leadership, cooperation, collaboration, and communication tactics [6].

The healthcare leaders need to be better guided about the concepts of understanding and implementation strategies especially during the pandemics for the best work culture setting for nurses in terms of teamwork, professional commitment, nurse behavior in practice, satisfaction, and leadership. Additionally, they require authoritative baseline information on the elements influencing nurses' work cultures as a result of the COVID-19 epidemic for academic scholars, politicians, and nursing decision-makers.

This cross-sectional study was conducted to have a better understanding of how the COVID-19 pandemic affected the workplace variables that affected the nursing work environment at SBAHC both before and after the pandemic. These relationships will provide a helpful assessment of the key driving forces of the system, norms, and behaviors and will determine the description and nature of work culture during difficult circumstances.

## Materials and methods

Study design and participants

A hospital-based cross-sectional study was conducted from January 2020 to December 2020, covering a period before the outbreak of COVID-19 and a period after. The study was conducted at SBAHC, Riyadh, Saudi Arabia, a 511-bed tertiary care hospital.The total number of nurses across all working units is around 439. The total targeted nursing units are eight including Inpatients and Outpatients. All nurses who have worked in hospitals for more than three months are eligible to participate; those who have worked there for less than three months, head nurses, and nurse trainees are not included. The expected response was estimated to be a minimum of 205 nurses considering the confidence interval is set to 0.95.

Data collection instrument and procedure

The survey questionnaire consisted of questions about general demographic data (age, gender, nationality, and marital status) and work-related characteristics such as educational status, work experience, language, primary role, and working unit, of nurses linked with the study. The item answer scale used is the 6-point Likert scale (strongly agree, agree, tend to agree, tend to disagree, disagree, strongly agree). The questions were distributed into five construct areas that reflect the overall nursing work culture concept. These areas included: 1. Leadership scale, including leadership strategy and leadership approach as subscales; 2. Satisfaction scale, including behaviour and practice as subscale; 3. Teamwork scale, including teamwork and communication as subscale; 4. Nurse behaviour in practice scale; and 5. Professional commitment scale, as shown in Table 1

**Table 1:** Study Survey Questionnaire

Q1.Nurse leaders are setting a defined standard of practice in nursing units.	□strongly agree □agree □ tend to agree □ tend to disagree □disagree □ strongly disagree
Q 2.Head Nurse/ Manager is monitoring staff compliance of the Standards task rules.	□strongly agree □agree □ tend to agree □ tend to disagree □disagree □ strongly disagree
Q 3.Nurse Leaders' strategies are involving Nursing in the internal governance of the hospital.	□strongly agree □agree □ tend to agree □ tend to disagree □disagree □ strongly disagree
Q 4.Nurses in our unit have Career development/clinical ladder opportunities.	□strongly agree □agree □ tend to agree □ tend to disagree □disagree □ strongly disagree
Q 5.There are enough support services resources to get the job done.	□strongly agree □agree □ tend to agree □ tend to disagree □disagree □ strongly disagree
Q6.There are opportunities for nurses to participate in policy decisions.	□strongly agree □agree □ tend to agree □ tend to disagree □disagree □ strongly disagree
Q7.Head Nurse/ Manager is explaining the standards of task rules for staff.	□strongly agree □agree □ tend to agree □ tend to disagree □disagree □ strongly disagree
Q 8.Head Nurse/Manager is highly visible and accessible to staff.	□strongly agree □agree □ tend to agree □ tend to disagree □disagree □ strongly disagree
Q 9.Head nurse/Manager of staff is supportive of the nurses.	□strongly agree □agree □ tend to agree □ tend to disagree □disagree □ strongly disagree
Q 10.Nurses have plenty of opportunities to discuss patient care problems with head nurse/ manager	□strongly agree □agree □ tend to agree □ tend to disagree □disagree □ strongly disagree
Q 11.Head Nurse/Manager use mistakes as learning opportunities, not criticism.	□strongly agree □agree □ tend to agree □ tend to disagree □disagree □ strongly disagree
Q 12. Nurses are satisfied with the nursing tasks assignment in our unit.	□strongly agree □agree □ tend to agree □ tend to disagree □disagree □ strongly disagree
Q 13. Nurses recommend our unit as a good place to work.	□strongly agree □agree □ tend to agree □ tend to disagree □disagree □ strongly disagree
Q14 Nurses in our unit are satisfied with the nursing unit Leadership	□strongly agree □agree □ tend to agree □ tend to disagree □disagree □ strongly disagree
Q 15.Nurses in our unit, satisfied with their jobs.	□strongly agree □agree □ tend to agree □ tend to disagree □disagree □ strongly disagree
Q 16.Nurses are satisfied with the culture of the workplace.	□strongly agree □agree □ tend to agree □ tend to disagree □disagree □ strongly disagree
Q 17.I am comfortable with my work schedule and hours.	□strongly agree □agree □ tend to agree □ tend to disagree □disagree □ strongly disagree
Q 18 The burden of work does NOT affect the overall work practice.	□strongly agree □agree □ tend to agree □ tend to disagree □disagree □ strongly disagree
Q 19. Staff help each other in daily tasks.	□strongly agree □agree □ tend to agree □ tend to disagree □disagree □ strongly disagree
Q 20.There is a respectful relationship among staff.	□strongly agree □agree □ tend to agree □ tend to disagree □disagree □ strongly disagree
Q 21 There is a trust relationship among staff	□strongly agree □agree □ tend to agree □ tend to disagree □disagree □ strongly disagree
Q 22 There is a collaborative relationship among Staff.	□strongly agree □agree □ tend to agree □ tend to disagree □disagree □ strongly disagree
Q 23 There is a good deal of teamwork among nurses in our unit.	□strongly agree □agree □ tend to agree □ tend to disagree □disagree □ strongly disagree
Q 24 Nurses work with ask each other to pitch in and help when things get busy.	□strongly agree □agree □ tend to agree □ tend to disagree □disagree □ strongly disagree
Q 25 Nurses communicate with support to each other.	□strongly agree □agree □ tend to agree □ tend to disagree □disagree □ strongly disagree
Q 26.There is good communication between nursing and physician to get the job done.	□strongly agree □agree □ tend to agree □ tend to disagree □disagree □ strongly disagree
Q 27.Staff communicate appropriately with patients and families.	□strongly agree □agree □ tend to agree □ tend to disagree □disagree □ strongly disagree
Q 28 Staff documentation of the care plan is well communicated to get the job done.	□strongly agree □agree □ tend to agree □ tend to disagree □disagree □ strongly disagree
Q 29.Staff use appropriate language with other staff	□strongly agree □agree □ tend to agree □ tend to disagree □disagree □ strongly disagree
Q 30.Nurses in our unit have good control over the work tasks.	□strongly agree □agree □ tend to agree □ tend to disagree □disagree □ strongly disagree
Q 31.Nursing efficiently Managing their role and responsibilities timing.	□strongly agree □agree □ tend to agree □ tend to disagree □disagree □ strongly disagree
Q 32.Nurses effectively carry out their roles and responsibilities.	□strongly agree □agree □ tend to agree □ tend to disagree □disagree □ strongly disagree
Q 33.Nurses are enthusiastically performing their roles and responsibilities.	□strongly agree □agree □ tend to agree □ tend to disagree □disagree □ strongly disagree
Q 34.There is a respectful behavior among nurses in our unit	□strongly agree □agree □ tend to agree □ tend to disagree □disagree □ strongly disagree
Q 35.There is an innovative and creative work practice in our unit.	□strongly agree □agree □ tend to agree □ tend to disagree □disagree □ strongly disagree
Q 36.I feel very loyal to the nursing profession.	□strongly agree □agree □ tend to agree □ tend to disagree □disagree □ strongly disagree
Q 37.For me, nursing is one of the best of all professions.	□strongly agree □agree □ tend to agree □ tend to disagree □disagree □ strongly disagree
Q 38.I am proud to tell others that I am part of this profession.	□strongly agree □agree □ tend to agree □ tend to disagree □disagree □ strongly disagree
Q 39 I really care about the nursing care profession.	□strongly agree □agree □ tend to agree □ tend to disagree □disagree □ strongly disagree

Question items are referenced for easy tracking and linkage. The survey was crafted utilizing google form software using a private email. A link was generated and shared with participants.

This survey was conducted first before the COVID-19 outbreak. The duration of data collection was completed within a three-week period, considering the schedule of the nurse’s duty and off time. The same survey was repeated after six months of the COVID-19 outbreak, without any changes in the questionnaire or participating group

Validity and reliability

The reliability and validity testing for the survey instrument was conducted through a trial of testing consistency. The questionnaire survey was used in a pilot study of 37 nurses, which indicated the suitability and stability of the instrument as a tool for the research study. The main reason for the piloting phase was to verify the item sequence, the practicability of the tool, and to add more clarity to the language to be more understandable to the participants.

Statistical analysis

Checked, cleaned, and coded data were entered in MS Excel version 365 for analysis. Descriptive statistics like frequencies, percentages, mean, and standard deviation were used to present data collected from the questionnaires. Comparison between the two groups was performed using t-tests. The two-sided tests were used, and p-value <0.05 represented a statically significant difference.

The study was approved by the institutional review board in Sultan bin Abdulaziz Humanitarian City (IRB review no.42-2021-IRB). Written informed consent was obtained from all participants. The study used anonymous data for analysis. The study was performed in accordance with the ethical standards laid down in the 1964 Declaration of Helsinki.

## Results

A total of 322 and 201 nurses completed the questionnaire before and after the COVID-19 outbreak respectively. The percentage of female nurses was found to be higher in both groups (71.1% and 75.1%). The age group (31- 40 years) showed the inclusion of the maximum number of nurses (52.2% and 56.7%). The percentage of married nurses was higher in both groups (52.5% and 52.7%). Most nurses (80.7% and 86.5%) had bachelor’s degrees. In both groups, the commonest nationality of nurses was Filipino (92.5% and 93.5%). The percentage of nurses with work experience of one to five years was 57.1% and 52.23%. There was a difference in the number of staff nurses and nurse assistants before and after COVID-19 (74.8% vs 83.0% and 25.2% vs 16.9%). When taking into consideration the percentage of nurses in each unit, there was a significant difference between the before and after groups in the case of General Rehab (7.4% and 16.4%), OPD (3.4% and 8.4%), Paediatric (27.0% and 8.4%) and Stroke Unit (7.1% and 19.9%). However, there was an increase in the number of nurses in the General Rehab, OPD, and Stroke Unit, but a decline in the number of nurses from the Paediatric Unit was seen (Table 2).

**Table 2:** Socio-Demographic Characteristics of Nurses before and after COVID-19

Variable	Category	Frequency N (%) 322	Category	Frequency N (%) 201	P-Value
BEFORE			AFTER		
Gender	Female	229 (71.1)	Female	151 (75.1)	0.31
	Male	93(28.9)	Male	50 (24.8)	0.30
Age	20-30 years	103 (32.0)	20-30 years	52 (25.8)	0.13
	31-40 years	168 (52.2)	31-40 years	114 (56.7)	0.31
	41-50 years	44 (13.7)	41-50 years	32 (15.92)	0.48
	> 50 years	7 (2.2)	> 50 years	3 (1.49)	0.56
Marital status	Single	149 (46.3)	Single	94 (46.76)	0.91
	Married	169 (52.5)	Married	106 (52.73)	0.96
	Others	4 (1.2)	Others	1 (0.49)	0.31
Educational status	Diploma	55 (17.1)	Diploma	22 (10.94)	0.05
	B.Sc. Nurse	260 (80.7)	B.Sc. Nurse	174 (86.56)	0.07
	M.Sc. Nurse	7 (2.2)	M.Sc. Nurse	5 (2.48)	0.88
Nationality	Filipino	298 (92.5)	Filipino	188 (93.53)	0.65
	Others	24 (7.4)	Others	13 (6.46)	0.66
Language	English as first language	87 (27.0)	English as first language	44 (21.89)	0.12
	English as 2nd language	235 (73.0)	English as 2nd language	157 (78.10)	0.19
Work experience at SBAHC	1-5 years	184 (57.1)	1-5 years	105 (52.23)	0.26
	6- 10 years	96 (29.8)	6- 10 years	68 (33.83)	0.33
	More than 10 years	42 (13.04)	More than 10 years	28 (13.93)	0.771
Primary Role	Staff Nurse	241 (74.8)	Staff Nurse	167 (83.08)	<0.05
	Nurse Assistant	81 (25.2)	Nurse Assistant	34 (16.91)	<0.05
Working unit	General Rehab	24 (7.4)	General Rehab	33 (16.41)	<0.05
	OPD	11 (3.4)	OPD	18 (8.95)	<0.05
	General Medical	30 (9.3)	General Medical	15 (7.46)	0.46
	Pediatric	87 (27.0)	Pediatric	17 (8.45)	<0.05
	Stroke	23 (7.1)	Stroke	40 (19.90)	<0.05
	Traumatic Brain injury	43 (13.35)	Traumatic Brain injury	17 (8.45)	0.08
	Women Health Unit	36 (11.2)	Women Health Unit	31 (15.45)	0.15
	Wound Care	18 (5.6)	Wound Care	9 (4.47)	0.57
	Others	50 (15.52)	Others	21 (10.44)	0.09

Tables 3 shows the means and standard deviations of the five research scales including subscales. There was a significant variation (p<0.005) in the response to the five scales used between the two groups. In all scales used, the mean score declined in the ‘after' group, clearly showing the effect of COVID-19 among the nurses.

**Table 3:** Relationship between Scale Score before and after COVID-19

Scale/Subscale		BEFORE	AFTER	P-value
Leadership	Total Leadership	5.11 ± 0.66	4.86 ± 0.12	<0.05
	Leadership Strategy	5.16 ± 0.63	4.89 ± 0.12	<0.05
	Leadership Approach	5.07 ± 0.72	4.90 ± 0.07	<0.05
Satisfaction	Total satisfaction	4.98 ± 0.77	4.56 ± 0.10	<0.05
	Job satisfaction	5.01 ± 0.78	4.64 ± 0.07	<0.05
	Job stress	4.97 ± 0.86	4.38 ± 0.03	<0.05
Teamwork	Total teamwork	5.21 ± 0.68	5.06 ± 0.06	<0.05
	Teamwork	5.25 ± 0.74	5.09 ± 0.03	<0.05
	Communication	5.18 ± 0.66	5.03 ± 0.07	<0.05
Nurse behavior in practice		5.11 ± 0.70	4.90 ± 0.05	<0.05
Professional Commitment scale		5.43 ± 0.63	5.28 ± 0.04	<0.05

Testing the association between demographics and nursing work culture scale domains, we found a significant difference (p<0.005) in all the groups. When comparing the leadership scale, we found a significant difference between the two groups (p<0.005) in terms of gender, marital status, education, and the primary role of nurses. Although there was no difference in the age group >50 years and the nurses with experience of >15 years, showing their leadership stability before and after COVID-19. When testing the leadership scale in different working units, all the units showed a significant difference except the Stroke Unit giving the impression that the nurses following routinely rehabilitation principles in their care with stroke rehabilitation skills have developed effective stroke care units (Table 4).

**Table 4:** Relationship between Demographic and Scale score

		Leadership			Satisfaction			Nurse Behavior in practice			Teamwork			Professional Commitment		
		Before	After	p	Before	After	p	Before	After	p	Before	After	p	Before	After	p
Gender	Female	5.11 ± 0.6	4.91 ± 0.6	<0.05	5.03 ± 0.7	4.68 ± 0.8	<0.05	5.12 ± 0.6	4.71 ± 0.6	<0.05	5.1 ± 0.5	3.32 ± 0.3	<0.05	5.37 ± 0.5	5.17 ± 0.4	<0.05
	Male	4.99 ± 0.9	4.86 ± 0.7	<0.05	4.87 ± 1	4.66 ± 0.8	<0.05	4.95 ± 1	4.69 ± 0.7	<0.05	5.01 ± 0.7	2.83 ± 0.4	<0.05	5.24 ± 0.8	5.07 ± 0.6	<0.05
Age (Y)	20-30	5.2 ± 0.5	4.81 ± 0.5	<0.05	5.06 ± 0.6	4.54 ± 0.8	<0.05	5.15 ± 0.6	4.61 ± 0.7	<0.05	4.99 ± 0.5	2.8 ± 0.8	<0.05	5.34 ± 0.5	5.06 ± 0.4	<0.05
	31-40	5.13 ± 0.6	4.96 ± 0.5	<0.05	4.98 ± 0.8	4.84 ± 0.5	<0.05	5.15 ± 0.6	4.77 ± 0.6	<0.05	5.02 ± 0.5	3.11 ± 0.3	<0.05	5.35 ± 0.5	5.08 ± 0.5	<0.05
	41-50	5.07 ± 0.7	4.84 ± 0.8	<0.05	5 ± 0.8	4.72 ± 0.8	<0.05	5.11 ± 0.7	4.73 ± 0.7	<0.05	4.98 ± 0.8	3.28 ± 0.4	<0.05	5.34 ± 0.7	5.03 ± 0.8	<0.05
	> 50	4.93 ± 1	4.83 ± 1	0.265	4.87 ± 1	4.71 ± 1	0.061	4.97 ± 1	4.7 ± 0.9	<0.05	4.91 ± 1	3.23 ± 0.5	<0.05	5.21 ± 1	4.99 ± 0.9	<0.05
Marital status	Single	5.04 ± 0.8	4.84 ± 0.6	<0.05	4.88 ± 0.9	4.61 ± 0.8	<0.05	4.98 ± 0.9	4.65 ± 0.7	<0.05	5.02 ± 0.5	2.84 ± 0.4	<0.05	5.2 ± 0.7	5.11 ± 0.5	<0.05
	Married	5.16 ± 0.5	4.97 ± 0.9	<0.05	5.06 ± 0.7	4.77 ± 0.6	<0.05	5.17 ± 0.5	4.77 ± 0.6	<0.05	5.12 ± 0.5	3.15 ± 0.5	<0.05	5.37 ± 0.5	5.15 ± 0.5	<0.05
	Others	5.13 ± 0.7	5.11 ± 0.7	0.74	5.05 ± 0.7	4.72 ± 0.7	<0.05	5.15 ± 0.6	4.72 ± 0.7	<0.05	5.06 ± 0.7	3.14 ± 0.7	<0.05	5.35 ± 0.6	5.08 ± 0.7	<0.05
Education	Diploma	5.11 ± 0.6	4.97 ± 0.4	<0.05	4.98 ± 0.7	4.72 ± 0.6	<0.05	5.08 ± 0.6	4.72 ± 0.6	<0.05	5.09 ± 0.5	3.14 ± 0.3	<0.05	5.26 ± 0.4	5.09 ± 0.5	<0.05
	B.Sc.	5.08 ± 0.6	4.9 ± 0.7	<0.05	5.13 ± 0.6	4.75 ± 0.6	<0.05	5.22 ± 0.5	4.75 ± 0.6	<0.05	5.01 ± 0.6	3.3 ± 0.3	<0.05	5.45 ± 0.5	5.08 ± 0.5	<0.05
	M.Sc.	4.98 ± 0.9	4.79 ± 1	<0.05	4.98 ± 0.9	4.71 ± 0.9	<0.05	5.09 ± 0.8	4.71 ± 0.9	<0.05	4.83 ± 1	3.24 ± 0.5	<0.05	5.33 ± 0.9	4.92 ± 1	<0.05
Experience (Y):	1-5	5.32 ± 0.4	4.92 ± 0.5	<0.05	5.06 ± 0.5	4.69 ± 0.7	<0.05	5.12 ± 0.5	4.72 ± 0.6	<0.05	5.37 ± 0.3	2.63 ± 0.3	<0.05	5.06 ± 0.5	5.13 ± 0.4	0.092
	6- 10	5.09 ± 0.6	4.86 ± 0.7	<0.05	4.79 ± 0.9	4.66 ± 0.8	0.094	4.98 ± 0.6	4.68 ± 0.7	<0.05	5.23 ± 0.6	3 ± 0.3	<0.05	5 ± 0.7	5.07 ± 0.7	0.249
	11- 15	5.05 ± 0.6	4.83 ± 0.8	<0.05	5.1 ± 0.8	4.63 ± 0.9	<0.05	4.28 ± 0.6	4.65 ± 0.8	<0.05	5.39 ± 0.6	3.23 ± 0.3	<0.05	4.99 ± 0.8	5.04 ± 0.8	0.519
	>15	4.79 ± 1.2	4.77 ± 0.9	0.84	4.93 ± 1.2	4.59 ± 0.9	<0.05	4.91 ± 1.2	4.61 ± 0.9	<0.05	5.24 ± 1.3	3.46 ± 0.4	<0.05	4.94 ± 0.9	4.99 ± 0.9	<0.05
Primary Role	Staff Nurse	5.09 ± 0.6	4.8 ± 0.5	<0.05	4.95 ± 0.8	4.7 ± 0.6	<0.05	5.07 ± 0.7	4.74 ± 0.6	<0.05	5.07 ± 0.5	2.94 ± 0.4	<0.05	5.33 ± 0.5	5.14 ± 0.4	<0.05
	Nurse Assistant	5.04 ± 0.8	4.94 ± 0.7	<0.05	4.93 ± 0.9	4.69 ± 0.8	<0.05	5.05 ± 0.8	4.7 ± 0.7	<0.05	5.03 ± 0.7	3.07 ± 0.4	<0.05	5.28 ± 0.7	5.09 ± 0.6	<0.05
Working unit:	General rehab	5.23 ± 0.4	4.92 ± 0.6	<0.05	4.93 ± 0.4	4.64 ± 1	<0.05	4.99 ± 0.4	4.54 ± 1.1	<0.05	5.03 ± 0.5	2.56 ± 0.2	<0.05	5.26 ± 0.3	5.09 ± 0.4	<0.05
	OPD	5.38 ± 0.7	4.57 ± 1.4	<0.05	5.17 ± 0.7	4.19 ± 1.4	<0.05	5.31 ± 0.6	4.13 ± 1.4	<0.05	4.71 ± 1.4	2.9 ± 0.2	<0.05	5.41 ± 0.4	4.74 ± 1.4	<0.05
	General Medical	5.04 ± 0.7	4.93 ± 0.5	<0.05	4.91 ± 0.9	4.98 ± 0.5	0.249	4.99 ± 0.6	4.97 ± 0.6	0.703	5.15 ± 0.6	2.85 ± 0.2	<0.05	5.25 ± 0.5	5.18 ± 0.6	<0.05
	Pediatric	5.06 ± 0.8	4.77 ± 0.8	<0.05	5.07 ± 0.9	4.47 ± 1.1	<0.05	5.2 ± 0.6	4.56 ± 0.8	<0.05	4.92 ± 0.8	3.22 ± 0.2	<0.05	5.37 ± 0.6	5 ± 0.7	<0.05
	Stroke	4.78 ± 1.4	4.67 ± 1.2	0.099	4.73 ± 1.4	4.5 ± 1.1	<0.05	4.75 ± 1.4	4.47 ± 1.2	<0.05	4.71 ± 1.3	3.12 ± 0.7	<0.05	5.06 ± 1.4	4.78 ± 1.3	<0.05
	Traumatic	4.8 ± 1.2	4.73 ± 1.2	<0.05	4.83 ± 1.2	4.68 ± 1.2	<0.05	4.83 ± 1.2	4.71 ± 1.2	0.267	4.97 ± 1.3	2.97 ± 0.6	<0.05	4.92 ± 1.2	5 ± 1.3	0.476
	Women Health	5.1 ± 0.4	4.89 ± 0.5	<0.05	4.98 ± 0.6	4.63 ± 0.6	<0.05	5.07 ± 0.5	4.62 ± 0.4	<0.05	5.01 ± 0.6	3.29 ± 0.2	<0.05	5.24 ± 0.4	5.02 ± 0.4	<0.05
	Wound care	4.96 ± 1.1	4.69 ± 1.3	<0.05	4.85 ± 1.2	4.49 ± 1.2	<0.05	4.93 ± 1.1	4.37 ± 1.2	<0.05	4.53 ± 1.2	3.33 ± 0.7	<0.05	5.26 ± 1.1	4.65 ± 1.2	<0.05
	Others	4.86 ± 1.2	4.74 ± 1.1	<0.05	4.85 ± 1.1	4.55 ± 1.1	<0.05	4.87 ± 1	4.44 ± 1	<0.05	4.57 ± 1	3.42 ± 0.7	<0.05	5.28 ± 2	4.68 ± 1	<0.05

Comparing the satisfaction scale, we found a significant difference between the two groups (p<0.005) in terms of all the demographic domains, except in the age group >50 years among the nurses having experience of six to 10 years, showing their satisfaction before and after COVID-19. In terms of working units, all the units showed a significant difference except the General medical. Although Teamwork and Professional Commitment scale showed a significant difference (p<0.005) between all the demographic domains, there was no significance found in the case of years of experience with professional commitment (Table 4).

## Discussion

In this study, nurses reacted with their perception of how they are viewing the workplace factors, as they are carrying over the strategies and system of work and transforming them into safe practice and performance. This was also observed in the study of Barbie et al. [7]. However, the findings here are presenting the positive perception of nurses toward the work practices before the pandemic and a change in the same perception after the pandemic. Before COVID-19, a set of questions on a range of scales reflecting five categories, namely leadership, satisfaction, teamwork, nurse behavior in practice, and professional commitment, were used to operationalize the quality of nurses' work surroundings. The same set of questions were repeated after the outbreak of the pandemic. The study of the nurse's responses to the scale/subscale demonstrated a high degree of agreement that work variables contribute positively to establishing a good nursing work culture and get affected due to any prevailing circumstances. This study was consistent with other studies where the overall psychological burden of COVID-19 among the nurses showed a great impact [8,9]. The uniqueness of this data is that the work factors discussed in this study are the key factors that outline how the nurses' work culture within a unit is being formulated. Each unit has a unique scope of services, and unit systems and strategies are being controlled by policies that provide the guidance of work within the team.

The first scale in this study was leadership, which serves as a mediator, supporter, and facilitator of front-line workers' work practices by providing each unit with a well defined and closely supervised scope of practice. The nurses find the opportunity to participate in decision-making, process developments and a professional development plan set by leaders [10]. Leaders' approach was also perceived well by nurses, as leaders are visible, accessible, and provide the opportunity of the learning environment and have given floor to nurses to adapt, improve, and innovate [11,12]. At SBAHC, nursing leadership had created many strategic projects to accommodate a good nursing culture for nurses. There were many programs, such as regular rounding, reporting system, issues escalating policies, open communication meetings with leaders, resource availability, and more.

Nurses here have opportunities to speak up, improve, and make effective innovations and changes. In this study, we found that leadership strategy encountered problems during the pandemic, and we analyze the mechanism that mediates between supporting leadership and nurses' well-being at work in order to determine how supportive leadership can improve nurses' well-being at work. While there is much to be proud of in nurse leadership, the pandemic has shown that there are several areas that should be enhanced to better serve patients and the larger nursing workforce [13]. Although there was a significant drop in leadership scores among nurses after the pandemic, there was no effect among those over the age of 50, which was consistent with the findings of Daly et al. who cited the decision to bring retired nurses back into practice to support the workforce, but noted that there was little discussion regarding how these retired nurses would reintegrate into the workforce [14].

Job satisfaction can range from extreme satisfaction to extreme discontent and is characterized as a combination of thoughts and beliefs people have about their current jobs, or an emotional response defining the degree to which people like their jobs. Job satisfaction has been found to have an impact on productivity, commitment, retention, and turnover. As a result, job satisfaction among health care workers has substantial consequences for care quality and health outcomes [15,16]. SBAHC has built a regular evaluation and monitoring system which is directed to assess the satisfaction of staff through regular yearly surveys. Besides, there is a department of people experience that has many activities that are aimed to improve the employees' work and life experience. However, the obstacles employees experience, such as higher risk of infection, fear of becoming sick and infecting family and loved ones, harder workloads, mental health burden, lack of support, and disruptions in work-life balance, likely contributed to a decreased job satisfaction score [17]. Corresponding to the same finding, this study had a similar impact on job satisfaction due to either poor preparedness for the pandemic or because the second survey was conducted immediately after the pandemic and at that time staff knew less about the disease, due to availability of fewer guidelines for management, and there was little promise of vaccines or effective treatments.

Teamwork is a critical work factor in shaping the work culture. Although there are a system and strategies that align staff to enhance teamwork and communication, the nurse’s internal factor of motivation and satisfaction is a drive to comply with working as a team. Interpersonal relationships, desires, and needs of nurses are essential to establishing good teamwork. These factors if not managed well, could lead to conflict and disrupt the work culture. Leaders must meticulously observe these drives to enhance collaboration and communication which assist in decreasing the stress level and intention to leave [18]. At SBHAC, nurses have reacted with a good level of teamwork and communication. This level is affected by the ongoing and support with many activities of enhancing relationships such as team building, a celebration of success, and recognition of efforts. Working with multinational nurses requires an additional effort to align the teamwork, cultural diversity, and communication principles. This is linked to the leader’s strategy to implement measures that create teamwork to enhance job performance and maintain safety [19]. However a clear effect of pandemic was seen in teamwork score as well, which could be due the implementation of preventive measures such as social distancing hindering the team meetings and discussion and also a frequent sick leave of the fellow teammate

The nurse’s professional commitment and loyalty is the surrounding frame of all other work factors, as it energizes the nurse's beliefs toward having attachment and belongings to the value and mission of the hospital. It’s a reminder for nurses that their profession's core value is being passionate and caring, besides, it is a humanitarian job. In most improvement strategies, leaders tend to use these reminders to enhance the power of performance within nurses. As reflected in the findings, nurses have scored a high percentage with no variances among the categories. This reflects that they are ready to have the base of the nursing work culture. In other means, nurses are prepared to communicate and collaborate, ready to engage, comply with the leadership guidance, and are empowered to innovations [20]. The level of professional commitment is one of the most important factors affecting nurses' turnover rate [21]. Previous studies have reported that as nurses' level of professional commitment increases, their intention to leave the profession decreases accordingly [22]. In this study, the score of Nursing Professional Commitment Scale was above average in the first assessment however, the pandemic puts professional commitment into a more fragile position. Increased workload, longer working hours, a more stressful working environment, insufficient medical and protective materials and disrupted social relationships has negatively affected professional commitment.

In the nursing units, the staff has developed a sense of accountability and responsibility that them to gain control over the work behavior that is extended of their compliance to system ethical principles. Nurses are viewing there is acceptable behavior that is contributing to building a structured work culture within the units. The respectful behavior among staff assists them to build a good team. However, this does not mean that there are no conflicts or misleading behaviors, but it reflects they have policies and control to resolve such issues. These variations must be captured and dealt with, before it impacts on a bigger negative culture which can adversely affect nursing care and patient outcomes, especially in the area of patient safety [7].The nurse’s behavior needs ongoing observation and guidance for the reason that shifting of behavior could happen at any time as a result of stress or unfair treatment.

The main limitation of this study was that the second assessment was done just after few months of pandemic when nurses where facing huge mental trauma. The study also misses the assessment of nurses after receiving the vaccine jabs to monitor improvement in workplace factors if any.

## Conclusions

The COVID-19 pandemic changed the entire nature of the hospitals. This was evident when the workplace factors influencing the nursing work environment were greatly influenced during the outbreak in Saudi Arabia. The findings of this study should be considered by nurse managers and leaders when drafting the policies and programs to reduce the negative impact of COVID-19 on nurse retention. It should also provide baseline information that will allow health authorities to prioritize training programs that will support nurses during difficult times. Additionally, hospital administrators should be sensitive to nurses' needs and demands in order to increase their compliance with changing working conditions and recognize the problems they encountered.
